# SARS-CoV-2 Doggybone DNA Vaccine Produces Cross-Variant Neutralizing Antibodies and Is Protective in a COVID-19 Animal Model

**DOI:** 10.3390/vaccines10071104

**Published:** 2022-07-09

**Authors:** Eric M. Mucker, Rebecca L. Brocato, Lucia M. Principe, Robert K. Kim, Xiankun Zeng, Jeffrey M. Smith, Steven A. Kwilas, Sungwon Kim, Helen Horton, Lisa Caproni, Jay W. Hooper

**Affiliations:** 1 Virology Division, United States Army Medical Research Institute of Infectious Diseases, Frederick, MD 21702, USA; eric.m.mucker.civ@mail.mil (E.M.M.); becky.brocato@nasa.gov (R.L.B.); lucia.m.principe.ctr@army.mil (L.M.P.); jeffrey.smith@fda.hhs.gov (J.M.S.); steven.a.kwilas.civ@mail.mil (S.A.K.); 2 Pathology Division, United States Army Medical Research Institute of Infectious Diseases, Frederick, MD 21702, USA; robert.k.kim2.mil@mail.mil (R.K.K.); xiankun.zeng.civ@mail.mil (X.Z.); 3 Touchlight Genetics, Ltd., London TW12 2ER, UK; sungwon.kim@touchlight.com (S.K.); helen.horton@touchlight.com (H.H.); lisa.caproni@touchlight.com (L.C.)

**Keywords:** doggybone DNA vaccine, nucleic acid, vaccine, SARS-CoV-2, COVID-19, immunogenicity, protection

## Abstract

To combat the COVID-19 pandemic, an assortment of vaccines has been developed. Nucleic acid vaccines have the advantage of rapid production, as they only require a viral antigen sequence and can readily be modified to detected viral mutations. Doggybone™ DNA vaccines targeting the spike protein of SARS-CoV-2 have been generated and compared with a traditionally manufactured, bacterially derived plasmid DNA vaccine that utilizes the same spike sequence. Administered to Syrian hamsters by jet injection at two dose levels, the immunogenicity of both DNA vaccines was compared following two vaccinations. Immunized hamsters were then immunosuppressed and exposed to SARS-CoV-2. Significant differences in body weight were observed during acute infection, and lungs collected at the time of euthanasia had significantly reduced viral RNA, infectious virus, and pathology compared with irrelevant DNA-vaccinated controls. Moreover, immune serum from vaccinated animals was capable of neutralizing SARS-CoV-2 variants of interest and importance in vitro. These data demonstrate the efficacy of a synthetic DNA vaccine approach to protect hamsters from SARS-CoV-2.

## 1. Introduction

Nucleic acid-based vaccines, more specifically mRNA-based vaccines for the COVID-19 pandemic, have recently gained notoriety. Strategies for overcoming the technical hurdles related to nucleic acid-based vaccines (i.e., low immunogenicity) are being developed with advances in formulation and more effective vaccine delivery systems [1,2,3,4,5,6]. Medical countermeasures, including vaccines and therapeutics, are proving imperative for ending the pandemic, warranting the continual evaluation of COVID-19 vaccine approaches.

Several DNA vaccines are under evaluation in clinical trials, such as the COVID-19 vaccine ZyCoV-D that is currently approved for emergency use in India [7,8,9,10,11,12]. Conventional plasmid DNA vaccines, such as ZyCoV-D, are typically made in *Escherichia coli* bacteria using antibiotic resistance gene based selection [13]. An alternative approach to developing plasmid DNA vaccines is ‘Doggybone™ closed linear DNA’ (dbDNA™), which is a covalently closed linear DNA construct that is enzymatically manufactured, not in bacteria [14,15,16]. This construct consists of the antigen-expressing cassette comprising regulatory sequences, such as a promoter and polyA tail, with closed and fully complementary ends. Relative to plasmid DNA, production of Doggybone DNA is cleaner, faster, and more scalable. Comparative analyses have been performed for plasmid and dbDNA™ vaccines targeting HIV [16], influenza [15], and HPV [14], showing equivalent humoral and cellular responses in small animal models, as well as in minipigs (unpublished data).

Here, we compared a conventional plasmid DNA vaccine (nCoV-S) to dbDNA™ vaccines containing the same spike protein sequence (dbDNAS) and a stabilized version (dbDNAS(ST)) administered by jet injection (JET). A chemically immunosuppressed animal model of severe COVID-19 disease was used, and wild-type Syrian hamsters were immunosuppressed with cyclophosphamide [17]. Additionally, the capacity of the vaccinated animal serum to neutralize SARS-CoV-2 or pseudotyped variants was assessed using serum from vaccinated animals. We found the nCoV-2-S(JET) plasmid vaccine performed similarly to the dbDNAS(ST-JET) vaccine, and all three vaccines had some level of cross-neutralizing activity.

## 2. Materials and Methods

### 2.1. Plasmid Construction

The construction of pWRG/nCoV-S(opt) was previously described. When administered via jet injection, this plasmid is called nCoV-S(JET). Additional plasmids for the PsVNA were constructed by the deletion of 21 amino acids from the COOH terminus of the full-length plasmids synthesized at Genewiz (South Plainfield, NJ, USA) for better incorporation into pseudovirions [5]. Those constructs are pWRG/CoV-S(opt)Δ21 (WA-1), pWRG/CA-S(opt)Δ21(California, also known as Epsilon), and pWRG/NY-S(opt)Δ21(New York, also known as Iota). These constructs were cloned into the Notl-BgIII site of the DNA vaccine vector pWRG. Additionally, a Δ21 truncated Beta construct (CodexDNA, San Diego, CA, USA) was synthesized and directly cloned into the pWRG backbone. For the Delta and Delta+ variants, a full-length spike plasmid was acquired from GenScript (Piscataway, NJ, USA) and truncated by PCR to create the Δ21 truncation, and it was then cloned into pWRG using NotI/BamHI.

### 2.2. dbDNA Construction

A sequence matching to the spike expression cassette from the pWRG/nCoV-S(opt) plasmid (including CMV, intron A, SARS-CoV-2 Wuhan Spike open reading frame, bovine growth hormone polyadenylation signal) [5] termed dbDNAS(JET) was synthesized and inserted into the Touchlight template backbone proTLx-K D3F2 at AflII and NheI. The resulting pDNA template comprised the above- described expression cassette flanked by two recognition/binding sites for the TelN protelomerase from *E. coli* phage N15. The synthesized spike expression cassette for the stabilized dbDNA construct was identical to the wild-type version except for 2956–2961 nucleotide bases of spike, resulting in amino acid changes K986P, V987P [18,19,20,21,22]. The dbDNA with the spike expression cassette was manufactured as previously described [14,23,24].

### 2.3. Animal Vaccinations

Wild-type (female, aged 11–13 weeks) hamsters (*Mesocricetus auratus*) were anesthetized by inhalation of vaporized isoflurane using an IMPAC6 veterinary anesthesia machine (VetEquip, Livermore, CA, USA). Fur over the semitendinosus and biceps muscles (left leg) was removed using electric clippers. A PharmaJet^®^ Tropis device (PharmaJet, Golden, CO, USA) was used to deliver either 0.2 or 0.05 mg of DNA diluted in PBS in a 0.1 mL volume. Specifically, the disposable syringe of the Tropis device was pressed against the skin, and the device was activated, resulting in the delivery of a liquid jet into the muscle and overlying tissues.

### 2.4. Other Animal Procedures

In addition to vaccination, the following procedures were conducted after anesthetizing the hamsters as described above: intranasal challenge of virus, cyclophosphamide (CyP) intraperitoneal injections, and nonterminal blood collection. Intranasal instillation of SARS-CoV-2 USA-WA-1/2020, Genbank accession #MT020880, was administered in a volume of 50 µL for the challenge dose of 1000 PFU. CyP treatment (pharmaceutical grade) consisted of an initial loading dose of 140 mg/kg on Day 3, followed by maintenance doses of 100 mg/kg on Days 1 and 5. Vena cava blood collection was limited to 7% of total blood volume per week. Terminal blood collection was performed by cardiac injection at the time of euthanasia. All work involving infected animals was performed in an animal biosafety level 3 (ABSL-3) laboratory.

### 2.5. SARS-CoV-2 Challenge Stock

An aliquot of the third passage of SARS-CoV-2 USA-WA-1/2020 was received from the CDC and propagated in ATCC Vero 76 cells (ATCC, #CRL-1587, 99% confluent) in EMEM containing 1% GlutaMAX, 1% NEAA, and 10% heat-inactivated fetal bovine serum (FBS) at an MOI of 0.01. Supernatant was collected from cultures exhibiting characteristic CPE and clarified by centrifugation (10,000× *g* 10 min). Clarified virus was subjected to the following specifications: identification by SARS-CoV-2 RT-PCR assay, quantification by agarose-based plaque assay, free from contaminants by growth of chocolate agar plates, endotoxin testing using Endosafe^®^ nexgen-PTS (Charles River, Wilmington, NC, USA), and mycoplasma using MycoAlert test kit (Lonza, Muenchensteinerstrasse, Switzerland), and genomic sequencing. Passage 5 virus was used for the hamster experiment.

### 2.6. SARS-CoV-2 D614G Variant Stock

SARS-CoV-2 virus isolate SPL20.017.30994 was isolated from a swab from a symptomatic 25-year-old male stationed on a U.S. Naval vessel. Upon genomic sequencing, the isolate was found to have 14 mutations (6 nonsynonymous) relative to the first case reported in Washington State (MT020880) and contained the D614G mutation in the spike protein. Virus was added to Vero 76 cells at an MOI of 0.01. Cells were then incubated for 1 h for virus adsorption and maintained in EMEM with 10% FBS. The infected cells were harvested after 48 hrs. The flask underwent a single freeze/thaw, and the supernatant was clarified at 1500× *g* for 10 min at 4 °C prior to vialing. Vials were stored at −70 °C until use. All stocks were titrated via plaque assays.

### 2.7. Viral RNA Assay

SARS-CoV-2 viral RNA was detected as previously described [17]. Briefly, approximately 200 mg of lung tissue was homogenized in Trizol and extracted, and the RNA concentration was normalized before performing RT-PCR. Primer/probe sequences, assay controls and conditions, as well as assay limits were utilized as previously reported.

### 2.8. Pseudovirion Production and Pseudovirion Assay (PsVNA)

Pseudovirions were produced exactly as described [5]. The SARS-CoV-2 PsVNA was performed including the modifications previously described [5]. All assays utilized serum from blood collected at the timepoints described in the Results section. An imputed value was calculated for samples titers below the assay limit by dividing the assay limit by the square root of 2 [25].

### 2.9. Plaque Assay

Quantification of infectious virus in lung homogenates was performed as previously described [17]. Briefly, virus was diluted in EMEM with 10% FBS and antibiotics then placed onto ATCC Vero 76 cell monolayers in 6-well plates. After 1 h at 37 °C in a 5% CO_2_ incubator, 3 mL of agarose (0.6% SeaKem ME agarose, EBME with HEPES, 10% heat-inactivated FBS, 100× NEAA, 1% pen/strep, 0.1% gentamycin, and 0.2% fungizone) was overlaid, and the plates were incubated for two days at 37 °C in the 5% CO_2_ incubator. We then added 2 mL of neutral red stain (0.6% SeaKem ME agarose in EBME, 5% neutral red, 5% FBS), which we incubated overnight, and the plaques were visualized and counted using a light box. Plaques were counted, and virus titers in plaque-forming units per milliliter were calculated.

### 2.10. Plaque Reduction Neutralization Test (PRNT)

The PRNT was performed as previously described [17]. Briefly, virus was combined with antibody and incubated 1 h at 37 °C and then adsorbed onto ATCC Vero 76 cell monolayers in 6-well tissue culture plates. After 1 h at 37 °C in a 5% CO_2_ incubator, the method of overlaying with agarose and staining with neutral red was identical to the plaque assay method described above. PRNT50 titers are the reciprocal of the highest dilution that results in a 50% reduction in the number of plaques relative to the number of plaques visualized in the media alone (no antibody) wells. An imputed value was calculated for samples titers below the assay limit by dividing the assay limit by the square root of 2 [25].

### 2.11. Hematology

A five-part CBC differential using whole EDTA was performed using aVETSCAN HM5 hematology analyzer. Sample species was set to DOG2 for analysis.

### 2.12. Preparation of Tissues for Histology

Tissues were fixed in 10% neutral buffered formalin, trimmed, processed, embedded in paraffin, cut at 5 to 6 µm, and stained with hematoxylin and eosin (H&E).

### 2.13. In Situ Hybridization

To detect SARS-CoV-2 genomic RNA in formalin-fixed, paraffin-embedded (FFPE) tissues, in situ hybridization (ISH) was performed using an RNAscope 2.5 HD RED kit (Advanced Cell Diagnostics, Newark, CA, USA) as previously described [26]. Briefly, forty ZZ ISH probes targeting SARS-CoV-2 genomic RNA fragment 21571-25392 (GenBank #LC528233.1) were designed and synthesized by Advanced Cell Diagnostics (#854841). Tissue sections were deparaffinized with xylene. The slides then underwent a series of ethanol washes, followed by a peroxidase blocking step. Next, the slides were heated in a kit-provided antigen retrieval buffer and digested by a kit-provided proteinase. Sections were exposed to ISH target probe pairs, incubated at 40 °C in a hybridization oven for 2 h, and then rinsed. The ISH signal was amplified using kit-provided Pre-amplifier and Amplifier conjugated to alkaline phosphatase and incubated with a Fast Red substrate solution for 10 min at room temperature. Fixed tissue sections were stained with hematoxylin, air-dried, and finally a coverslip was applied.

### 2.14. Statistical Analyses

Statistical analyses were completed using GraphPad Prism 8. An ordinary one-way ANOVA with Tukey multiple comparisons was used to analyze immunogenicity, viral RNA, and infectious virus. Weight was analyzed using a two-way ANOVA with Dunnett’s multiple comparisons. In all analyses, *p* < 0.05 was considered statistically significant.

## 3. Results

### 3.1. Immunogenicity of SARS-CoV-2 DNA Vaccines Administered by Jet Injection in Syrian Hamsters

**Figure 1:**
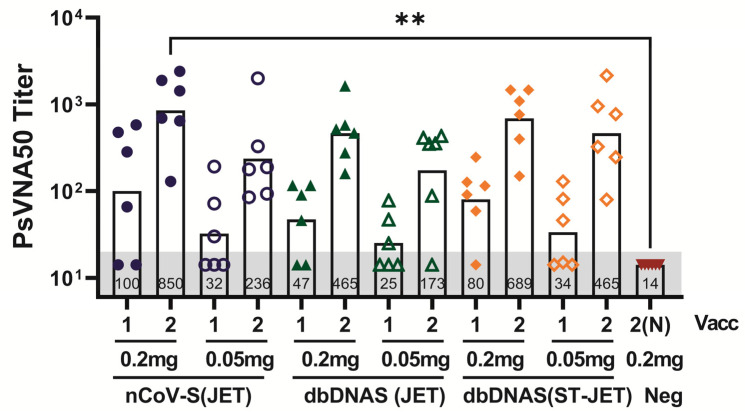
Immunogenicity of DNA vaccines administered by jet injection in Syrian hamsters. Hamsters were vaccinated with either 0.2 mg or 0.05 mg of either nCoV-S(JET), dbDNAS(JET), dbDNAS(ST-JET), or 0.2 mg negative control DNA (*n* = 6 per group) on Weeks 0 and 3. Serum collected three weeks following 1st vaccination (vacc) or two weeks after the 2nd vacc was analyzed by WA-1 PsVNA, and is presented as PsVNA50 titers. An imputed value [25] of 14.1 was used for samples with titers below the assay limit of 20 (grey shade). Annotations within each bar indicate geometric mean titers (GMTs). Individual symbols represent titers from a single hamster. Significant results of comparisons between vaccinated group titers and the control (2N) group titers, as well as comparisons between group titers generated from the same number of vaccinations (i.e., 1 vs. 1 and 2 vs. 2 (or 2N) are shown (** *p* < 0.01).

A plasmid DNA and a dbDNA vaccine were made using identical SARS-CoV-2 spike sequences. These vaccines, nCoV-S(JET) and dbDNAS (JET), enabled a direct comparison of the immunogenicity of plasmid versus linear DNA vaccines. Hamsters were vaccinated with either 0.2 or 0.05 mg of vaccine by jet injection, and immunogenicity was determined by a SARS-CoV-2 PsVNA following the first and second vaccinations. With the exception of a single hamster in the dbDNAS (JET) group dosed at 0.05 mg, all hamsters had neutralizing titers after the second vaccination. Overall, the highest neutralizing levels were observed in the nCoV-S(JET) or dbDNAS(ST-JET) groups, regardless of number of vaccinations or dose administered (Figure 1). There were statistically significant increases in PsVNA titer following the second nCoV-S(JET) relative to the mock-vaccinated control (Figure 1). Similar PsVNA50 neutralizing antibody titers were detected following each vaccination by dose level.

### 3.2. Protective Efficacy of DNA Vaccines in Immunosuppressed SARS-CoV-2 Exposed Hamsters

**Figure 2:**
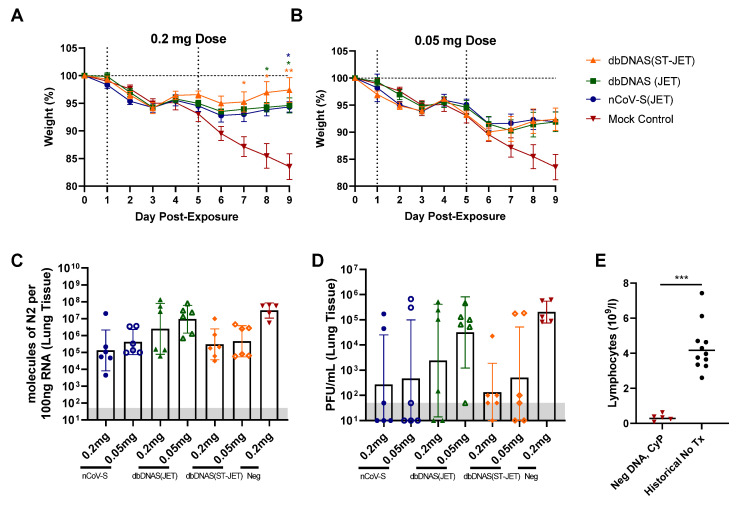
Protective efficacy of DNA vaccines in SARS-CoV-2 hamsters. Vaccinated hamsters from Figure 1 were treated with cyclophosphamide and exposed to SARS-CoV-2. (**A**,**B**) Weights were monitored daily. Individual hamster values are shown as symbols. Asterisks above and below indicate statistical significance compared with negative DNA vaccine controls and are color coated for weights to designate group. Lung tissue was analyzed by (**C**) real-time RT-PCR and (**D**) plaque assay. (**E**) Lymphocyte counts were assessed from whole blood collected at 9 dpi and compared with historical, untreated hamster controls (***, *p* < 0.001; **, *p* < 0.01; *, *p* < 0.05). The assay limit for panels C and D is shaded grey.

The protective efficacy of nCoV-S(JET), dbDNAS(JET), and dbDNAS(ST-JET) vaccines was assessed using the immunosuppressed SARS-CoV-2 hamster model [17]. Although hamsters have been shown to be adequate models of SARS-CoV-2 infection [5,17,27,28], the immunosuppressed model was utilized because it is a more stringent disease model, as it exacerbates and prolongs clinical and pathological manifestations while still using a low dose of inoculum [17]. Vaccinated hamsters were immunosuppressed with cyclophosphamide and exposed to 1000 PFU SARS-CoV-2 by the intranasal route. Statistically significant weight differences were observed for all groups vaccinated with the 0.2 mg dose at Days 7–9 (dbDNAS(ST-JET) group), Day 8 and 9 (dbDNAS (JET) group) or just Day 9 (nCoV-S(JET) group) post-injection (Figure 2A,B). Lung tissue collected at 9 dpi was assayed for viral RNA and infectious virus by real-time RT-PCR (Figure 2C) and plaque assay (Figure 2D), respectively. Both viral RNA copies and infectious viral geometric mean values from lung samples were (nonstatistically) decreased relative to the negative DNA control. Hematology was assessed to confirm reduced lymphocyte counts (Figure 2E). Together, these data indicate that 0.2 mg of either nCoV-S(JET) or dbDNAS(ST-JET) has a protective effect in SARS-CoV-2-exposed hamsters, resulting in significantly reduced weight loss, viral RNA, and infectious virus detected in the lungs. The protective effect was markedly less in hamsters vaccinated with 0.05 mg of either nCoV-S(JET) or dbDNAS(ST-JET) vaccines, or either administered dose of dbDNAS(JET).

### 3.3. Lung Pathology of SARS-CoV-2 DNA Vaccinated Hamsters Following Virus Exposure

**Figure 3:**
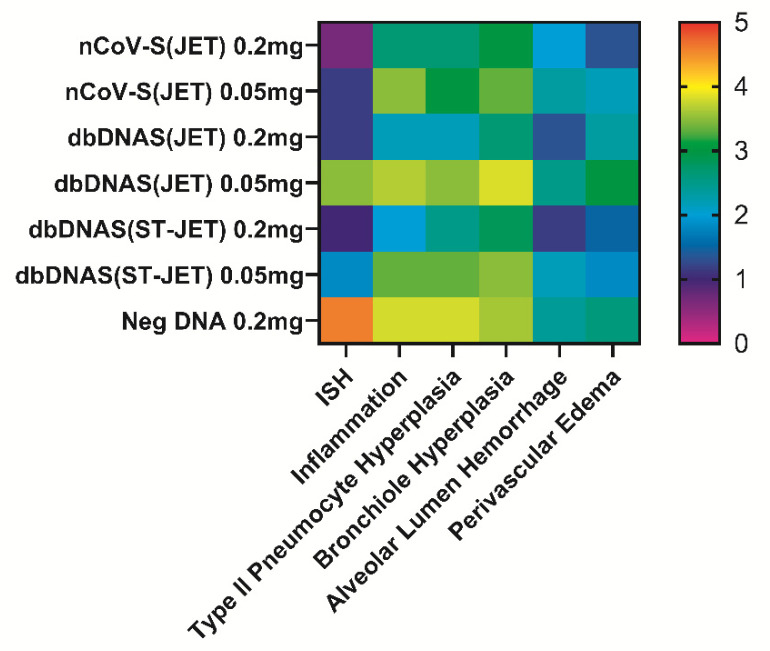
Heat map depicting degree of ISH labeling and severity of histopathological lesions observed in DNA-vaccinated hamsters. ISH slides of lung sections were scored by degree of labeling: 0 = no labeling, 1 = rare or <10% of cells, 3 = multifocal dense labeling or 10–60% of cells, and 5 = greater than 60% of cells labeled. H&E slides of lung sections were scored by severity: 0 = no detected pathology, 1 = minimal, 2 = mild, 3 = moderate, 4 = marked, and 5 = severe.

**Figure 4:**
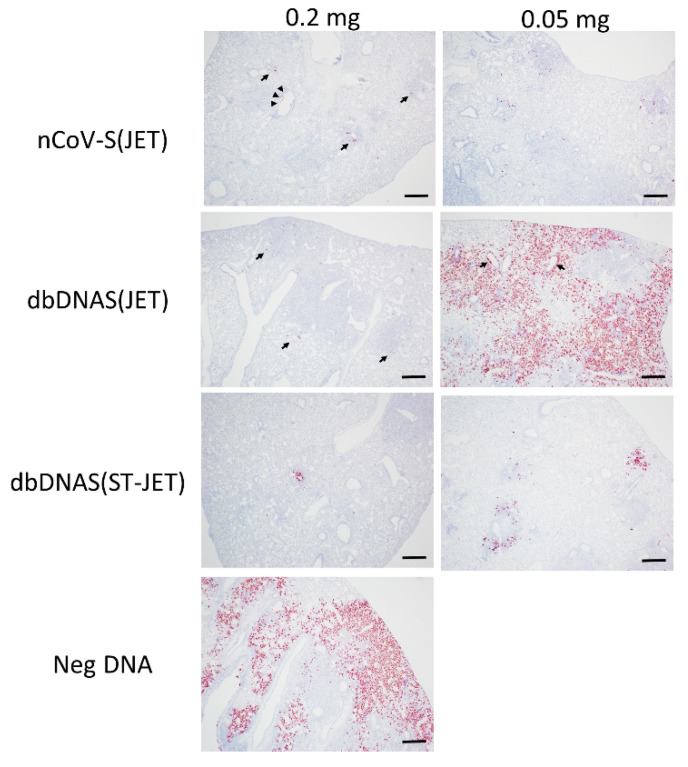
SARS-CoV-2 ISH labeling of FFPE lung tissues from DNA-vaccinated hamsters. Lung sections from 0.2 and 0.05 mg DNA-vaccinated hamsters show SARS-CoV-2 genomic RNA in red. Arrows in nCoV-S(JET) and dbDNAS(JET) 0.2 mg images indicate rare positive labeling within the section, and arrowheads indicate labeling of intraluminal bronchiolar cellular debris. Arrows in dbDNAS 0.05 mg indicate multifocal dense positive labeling within areas of inflammation and bronchiolar epithelium. Scale bars = 400 microns.

All hamsters were euthanized at 9 dpi, and lung tissue from necropsied animals was formalin-fixed, paraffin-embedded, and evaluated by histopathology and in situ hybridization (ISH). The specific lesions observed in immunosuppressed hamsters exposed to SARS-CoV-2 have been described previously [5,17]. The severity of histopathological findings in the lungs (Figure 3) and extent of the ISH labeling (Figure 3 and Figure 4) corroborated what we observed in RT-PCR and plaque assays from lung homogenates. Hamsters vaccinated with 0.2 mg of either nCoV-S(JET), dbDNAS(JET), or dbDNAS(ST-JET) had reduced SARS-CoV-2 genomic RNA by ISH (Figure 4) and reduced incidence and severity of interstitial pneumonia, heterophilic and histiocytic inflammation, hyperplasia (type II pneumocyte, bronchial, and/or bronchiolar epithelium), alveolar hemorrhage, and perivascular edema (Figure 3).

### 3.4. Serum Neutralization Efficacy against Novel SARS-CoV-2 Variants

**Figure 5:**
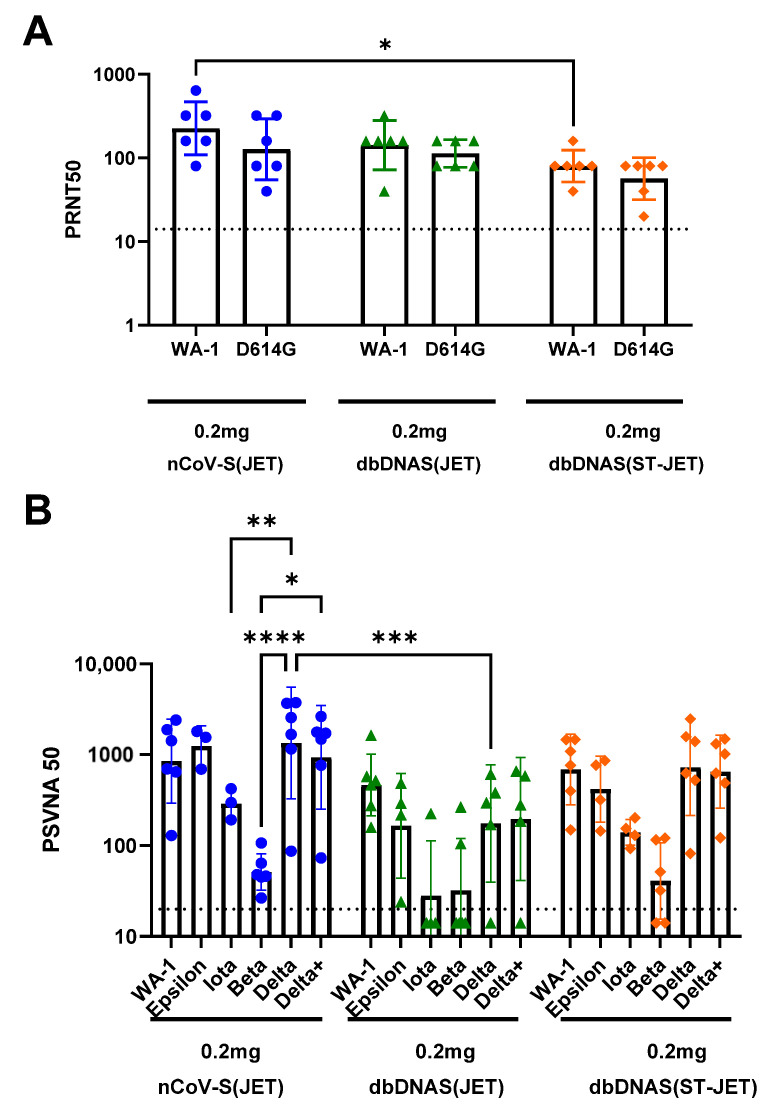
Cross-variant neutralization of serum from hamsters vaccinated with plasmid or Doggybone DNA vaccines. (**A**) Prechallenge serum from hamsters that received two 0.2 mg vaccinations of nCoV-S(JET), dbDNAS(JET), or dbDNAS(ST-JET) was used to perform Washington-1 (WA-1) and D614G variant live-virus PRNT. (**B**) Prechallenge sera were evaluated using Epsilon, Iota, Beta, Delta, and Delta+ pseudovirion neutralization assays. Individual animal titers (symbols) as well as geometric mean and standard deviations are shown for each group. Statistical significance (**** *p* < 0.0001; *** *p* < 0.001; ** *p* < 0.01; * *p* < 0.05) between variants and within vaccine groups, and same variants between vaccine groups are also shown. An imputed value [25] of 14.1 was used for samples with titers below the assay limit of 20 (dotted line).

Mutations in the viral genome are arising with new variants being identified as the pandemic progresses [29,30]. As SARS-CoV-2 variants are becoming more prevalent in human infections, it is necessary to determine if the polyclonal antibody responses to DNA vaccination (nCoV-S(JET), dbDNAS(JET), and dbDNAS(ST-JET)) have cross-neutralizing efficacy. The D614G mutation in the spike protein was one of the first identified substitutions [31,32]. A PRNT was performed using the Week 5 sera against the D614G variant virus. Serum from animals vaccinated with nCoV-S(JET) had the highest group neutralizing titers for both WA-1 and D614G variant viruses, but, with the exception of nCoV-S(JET) and dbDNAS(ST-JET) neutralization of WA-1 (*p* = 0.0159), there were no other statistical differences between variants within a vaccinated group or the same variant between groups (Figure 5A).

Additional variants Beta, Iota, and Epsilon, along with more current variants of concern, Delta and Delta+ (Delta with K417N substitution), were also used to assess the neutralizing capacity of sera from hamsters vaccinated with a high dose of either nCoV-S(JET), dbDNAS(JET), or dbDNAS(ST-JET) vaccines using variant virus pseudovirion neutralization assays (Figure 5B). All three vaccines elicited cross-neutralizing antibodies against all variants tested. However, the response to Beta was noticeably, but not significantly, reduced relative to WA-1, and the response to Iota was somewhat reduced. The dbDNAS(JET) cohort response was lower than that of the other two cohorts, only one animal was positive for anti-Iota antibodies, and only two were positive for anti-Beta antibodies. The nCoV-S(JET) samples neutralized Delta and Delta+ variants statistically differently than the Beta variant. Differences between the neutralization of Delta and Iota variants were also significant within the nCoV-S(JET) group. The only statistical difference between vaccine groups was the ability of nCoV-2(JET) and dbDNAS(JET) samples to neutralize the Delta variant. The responses against Epsilon, Delta, and Delta+ were similar within a cohort (Figure 5B).

## 4. Discussion

In this study, we compared a conventional plasmid vaccine, nCoV-S(JET), with a doggybone linear DNA vaccine. One doggybone DNA vaccine, dbDNAS(JET), provides a direct comparison to the plasmid DNA vaccine as the sequences used in these vaccines are identical. No statistical differences in immunogenicity were observed at either the 0.05 or 0.2 mg dosage, making these vaccines comparable. However, once hamsters were exposed to SARS-CoV-2, higher, but not statistically significant, levels of viral RNA and infectious virus were detected in the lungs of dbDNAS hamsters, most prominently in the dbDNAS 0.05 mg vaccination group. The stabilized version of the doggybone DNA vaccine, dbDNAS(ST-JET), had comparable immunogenicity and similar protective efficacy to nCoV-S(JET) in cyclophosphamide-immunosuppressed hamsters, the 0.2 mg dbDNAS(ST-JET) having a slight advantage based on changes in weight. That is, the dbDNAS(ST-JET) group had statistically significant differences in weight loss relative to the control group on the last three days of the study, whereas nCoV-S(JET) was significant on the last day alone. This difference did not translate into any other significant differences in other correlates, such as viral lung burden or pathology.

Although our animal efficacy are promising, there are limits to our modeling system. More specifically, the model likely reflects disease in a subpopulation of people (e.g., immunosuppressed). In hamsters, there is little to no adaptive immune response that may enhance or detract from the efficacy of a specific vaccine and paint an incomplete picture of a vaccinated host’s response to infection. As with all vaccine testing, future studies should include the use of multiple models.

Class I fusion proteins, such as the spike surface glycoprotein from betacoronaviruses and F protein from RSV, are major vaccine targets for the induction of protective neutralizing antibodies. Antibodies raised against the prefusion conformations are more efficacious at neutralizing free virus, and the use of mutations to stabilize this conformation has been shown to improve neutralizing antibody titers against multiple viruses. This is most profoundly seen in RSV-F, which is highly unstable in its prefusion form without the stabilizing double proline mutation [18,19,20,21,22,33,34]. In our study, the inclusion of the stabilizing mutations increased the immunogenicity and protective efficacy of the dbDNAS(ST-JET) relative to the unmodified spike sequence in dbDNAS(JET) vaccine. Similar to dbDNAS(JET), the plasmid DNA construct nCoV-S(JET) did not contain the mutations, but still performed similarly to the stabilized doggybone construct (dbDNAS(ST-JET)) and was more immunogenic and protective than dbDNAS(JET), which contains the exact sequence as nCoV-S(JET). This suggests that the vaccine may have some construct-related advantages that would warrant further investigation, specifically using stabilized spike pDNA compared with dbDNAS(ST-JET).

The cross-variant neutralization study revealed the similar neutralizing capacity of the sera from hamsters immunized with either nCoV-S(JET), dbDNAS(JET), or dbDNAS(ST-JET) against WA-1, Epsilon, Delta and Delta+. However, the neutralizing capacity of the sera was noticeably lower against the Beta variant. The Beta variant was first documented in South Africa in May 2020 and linked with increased risk of hospitalization and death [35]. Abu-Raddad et al. reported that two doses of the Pfizer vaccine showed less than 75% effectiveness against Beta-variant infection, compared with 89.5% effectiveness against the Alpha variant [36]. Additionally, analysis of the phylogenetic relationships between SARS-CoV-2 variants revealed that the Beta variant is evolutionarily distant from the U.S. WA-1 strain, unlike the Alpha and Delta variants [37]. Specifically, the phylogenetic analysis using NJ/JC method revealed that the U.S. WA-1 and Delta variants are branched under the same node, neighboring the Alpha and Omicron variants, whereas the Beta and Gamma variants evolved and split earlier from the main node [37]. The phylogenetic analysis corroborates the neutralization data, demonstrating that increased divergence of the spike protein within each variant correlates to reduced neutralization. Unfortunately, at the time of these studies, Omicron was not appreciably circulating, and we were unable to include it in our assays.

DNA vaccines have several advantages, including stability (minimal cold-chain requirements), safety, and increased immunogenicity with administration by needle-free delivery devices [4,38,39,40,41]. However, traditional pDNA manufacture is a major bottleneck [42]. Synthetic linear DNA overcomes the manufacturing bottlenecks, enabling production of grams of GMP dbDNA within days using benchtop equipment. Furthermore, the elimination of bacterial resistance genes provides an additional degree of safety, and also makes the resulting dbDNA construct smaller than a matched pDNA sequence, offering an additional copy number advantage. Thus, dbDNA is well-positioned to contribute to rapid pandemic response efforts.

## 5. Conclusions

The most effective way to end the pandemic is through the development of medical countermeasures to combat SARS-CoV-2 infection and disease. The rise of the variants has also illustrated the need for the rapid evolution of vaccines, a niche that is well-suited for nucleic-acid-based vaccines. Herein, we compared two DNA vaccine approaches: a conventional plasmid DNA vaccine and a novel, synthetic, linear DNA vaccine. Both vaccines were protective in a small animal model of severe disease. Moreover, serum from animals vaccinated with SARS-CoV-2 DNA vaccines retained a level of neutralization against multiple emerging variants.

## 6. Patents

J.W.H. is inventor on USG provisional SARS2 DNA vaccine patent application.

## Data Availability

All data are available upon request. Unique materials used in this study are available from the corresponding author by Material Transfer Agreement.
